# Design, Synthesis of Hydrogen Peroxide Response AIE Fluorescence Probes Based on Imidazo [1,2-a] Pyridine

**DOI:** 10.3390/molecules29040882

**Published:** 2024-02-16

**Authors:** Luan Tong, Yulong Yang, Likang Zhang, Jiali Tao, Bin Sun, Cairong Song, Mengchen Qi, Fengqing Yang, Mingxia Zhao, Junbing Jiang

**Affiliations:** 1 Department of Veterinary Medicine, Shanxi Agricultural University, Jinzhong 030801, China; tongluan0101@163.com (L.T.);; 2 Department of Mining Engineering, Shanxi Institute of Technology, Yangquan 045000, China; 3 Yangquan Technology Innovation Center of Carbon Dioxide Capture, Utilization and Storage, Shanxi Institute of Technology, Yangquan 045000, China

**Keywords:** hydrogen peroxide, AIE, fluorescence probe, imidazo [1,2-a] pyridine derivative

## Abstract

Hydrogen peroxide (H_2_O_2_), a significant member of reactive oxygen species, plays a crucial role in oxidative stress and cell signaling. Abnormal levels of H_2_O_2_ in the body can induce damage or even impair body function, leading to the development of certain diseases. Therefore, real-time monitoring of H_2_O_2_ in living cells is very important. In this work, the aggregation-induced emission fluorescence probe 2-(2-((4-(4,4,5,5-tetramethyl-1,3,2-dioxaborolan-2-yl) benzyl) oxy) phenyl) imidazo [1,2-a] pyridine (**B2**) was designed and synthesized, which enables the long-term tracing of H_2_O_2_ in living cells. The addition of H_2_O_2_ to probe **B2** results in a dramatic fluorescence enhancement around 500 nm. Notably, **B2** can visualize both exogenous and endogenous H_2_O_2_ in living cells. The synthesis method for **B2** is simple, has a high yield, and utilizes readily available materials. It exhibits advantages such as low toxicity, photostability, and good biocompatibility. Consequently, the developed fluorescent probe in this study has great potential as a reliable tool for determining H_2_O_2_ in living cells.

## 1. Introduction

Reactive oxygen species (ROS), which include H_2_O_2_, O_2_^−^, OH^−^, O_3,_ and ^1^O_2_, possess higher redox activity compared to ordinary oxygen atoms [1]. Hydrogen peroxide (H_2_O_2_) is produced when NADPH oxidase complexes are activated by cytokines, neurotransmitters, and peptides that promote cell growth. It acts as a second messenger in signal transduction during host defense, immune response, and pathogen invasion [2]. However, abnormal production or accumulation of H_2_O_2_ can disrupt protein structure and function, resulting in oxidative cell damage and causing diseases such as cancer, cardiovascular disease, and neurological disorders. There are numerous ways to detect H_2_O_2_ today, including chemiluminescence [3], electrochemistry [4], and spectrometry analysis [5]. When compared to traditional detection methods, fluorescent probes paired with optical imaging technology offer the advantages of simplicity, high sensitivity, and non-invasiveness. Consequently, this approach has gained widespread use, such as detecting macromolecules, screening drugs, identifying tumors, and studying gene expression in various applications [6,7,8,9,10,11,12,13].

Currently, there are seven types of recognition groups used for the detection of H_2_O_2_. These groups are based on aryl borate [14], benzoyl [15], sulfonate [16], selenium oxide [17], acetyl [18], Payne/Dakin reaction [19], and methenonitrile [20]. Boric acid or borate esters are commonly utilized as superior components in H_2_O_2_ reactions. In 2004, Chang’s group [21] reported the H_2_O_2_ probe PF1; they introduced a pinacol borate into a fluorescent probe. It was a new type of optical probe for intracellular imaging of H_2_O_2_ in living biological samples at the 3′ and 6′ positions of a xanthenone scaffold containing two boronic ester groups as a response moiety. It is worth mentioning that PF1 utilizes a boronate deprotection mechanism to provide unprecedented selectivity and optical dynamic range for detecting H_2_O_2_ in an aqueous solution. Furthermore, the probe PF1 was applied to the fluorescence imaging of exogenous H_2_O_2_ in HEK293T cells, and good results were obtained. In 2016 and 2017, Yibing Zhao’s group [22] designed two lysosomal-targeted H_2_O_2_ probes. The probe is a pH-switchable fluorescent H_2_O_2_ probe that responds sequentially to intracellular H_2_O_2_. The amphiphilic and nucleophilic nature of the interaction between H_2_O_2_ and the borate probe enables advantageous hydrolysis. Compared to other ROS probes with non-specific oxidation mechanisms, the specific interaction between H_2_O_2_ and borate exhibits higher selectivity [23,24,25].

Numerous fluorescent probes based on small-molecule fluorophores have been developed for the detection of H_2_O_2_, including rhodamine [26], coumarin [27], naphthalimide [28], and BODIPY [29]. In 2014, Shijun Shao’s group [30] reported a case of the BODIPY derivatives probe, which was designed and synthesized via the N-alkylation reaction of meso-(4-pyridinyl)-substituted BODIPY and showed a novel water-soluble “turn-on” fluorescent probe for the discrimination of H_2_O_2_. In 2020, Tian’s group [31] connected a naphthalimide derivative to a rhodamine derivative and developed a two-photon fluorescence probe TFP-based lifetime, which offered real-time imaging and the simultaneous determination of mitochondrial H_2_O_2_ and ATP changes in two well-separated fluorescence channels without spectral crosstalk. However, these probes exhibit small Stokes shifts. Conversely, fluorescent probes with large Stokes shifts minimize crosstalk between excitation light and emission light, leading to a significant improvement in the signal-to-noise ratio [32].

According to the application requirements of the next-generation probes, fluorescent probes with large Stokes shifts can realize accurate imaging and sensing. Unlike traditional fluorescence, which exhibits aggregation-induced quenching (ACQ), molecules with aggregation-induced emission (AIE) have the advantage of almost no emission in a weak solution but high emission in an aggregation state [33]. AIE serves as a selective “turn-on” fluorescent probe for bioanalysis [34,35,36,37] and enables long-term monitoring of biological processes due to its exceptional properties, including significant Stokes shifts, photostability, and accurate signal output at high concentrations [38]. Thus, developing fluorescent probes with AIE properties is very important due to the significant academic value and biological applications of AIE materials.

This study aimed to capitalize on the properties of AIE by developing a fluorescent probe that consisted of an imidazole [1,2-a] pyridine group as the fluorescent moiety and a borate ester as the reactive group for H_2_O_2_. The newly constructed probe, 2-(2-((4-(4,4,5,5-tetramethyl-1,3,2-dioxaborolan-2-yl) benzyl) oxy) phenyl) imidazo[1,2-a] pyridine (**B2**), was designed for the selective detection of H_2_O_2_, allowing for on-site sensing and long-term tracking. The incorporation of the borate ester moiety in **B2** enables highly sensitive detection of H_2_O_2_. Upon reaction with H_2_O_2_, the boronate group of **B2** undergoes cleavage, leading to the formation of the **B2**-OH fluorophore. The fluorescent probe exhibits a pronounced fluorescence turn-on signal, reliably detecting H_2_O_2_ in the fluorescence imaging of A549 cells. Based on the collective findings, this probe demonstrates great potential as a sensor for living-cells detection of H_2_O_2_ and serves as a valuable reference for the development of highly sensitive fluorescent probes.

## 2. Results and Discussion

### 2.1. Synthesis of Probe **B2**

**Scheme 1:**
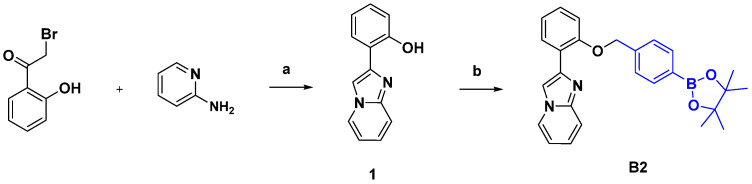
(**a**) Anhydrous ethanol, sodium bicarbonate, reflux, 2 h, 72.8%; (**b**) potassium carbonate, anhydrous acetonitrile, reflux, 6 h, 73.3%.

**Figure 1:**
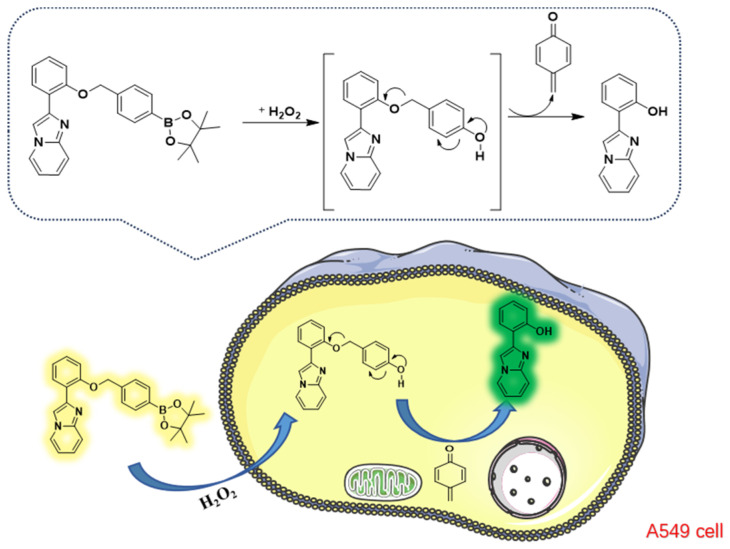
The principle of ESIPT-based fluorescent probe **B2** detecting H_2_O_2_.

The synthesis of probe **B2** is outlined in Scheme 1. The reaction of 2-hydroxyacetophenone 2-aminopyridine morpholine in EtOH was refluxed to obtain compound **1**. In the second step, compound **1** is reacted with 4-(Bromomethyl) benzene boronic acid pinacol ester in anhydrous acetonitrile, resulting in the formation of probe **B2** with a yield of 73.3%. In this work, imidazole [1,2-a] pyridine is synthesized and discovered as a fluorophore for the first time. The boronic ester group is selected as the recognition moiety to detect H_2_O_2_ because it exhibits a “turn-on” fluorescent signal that can be easily distinguished, which is illustrated in Figure 1. The characterizations of compound **1** and probe **B2** were confirmed using ^1^H NMR, ^13^C NMR, and a mass spectrum (MS), as shown in Figures S1–S6 in the supporting information. The CheckCIF report of compound **1** is shown in the supporting information. Furthermore, the probe is soluble in common solvents such as ethyl acetate, dichloromethane, methanol, and acetonitrile.

### 2.2. Optical Characterizations of Probe **B2**

**Figure 2:**
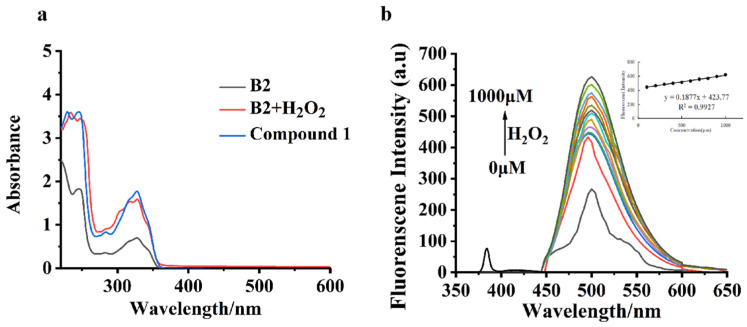
(**a**) UV–Vis absorption spectra of compound 1 and **B2** and **B2** after addition of H_2_O_2_; (**b**) fluorescence emission spectra of probe **B2** (5 μM) following incubation with an increasing concentration of H_2_O_2_ (30, 50, 100, 150, 200, 300, 450, 500, 600, 700, 800, 900, and 1000 µM). The experiments were repeated three times, and the data are shown as mean (±S.D.).

At first, the absorption spectra of **B2** before and after adding H_2_O_2_ were measured. As described in Figure 2a, in the UV–Vis absorption, which was studied in the absence of H_2_O_2_, an absorption of **B2** at 325 nm is seen; however, after adding H_2_O_2_, the absorption peaked at a 325 nm strength. As shown in Figure S7, **B2** was weak when excited at 384 nm (Φ = 0.09), while a significant increase in fluorescence was observed at 500 nm after adding H_2_O_2_. The **B2** did not emit fluorescence at 500 nm, suggesting that the phenyl boronic acid pinacol ester group of the probe **B2** quenched the fluorescence of compound **1** (Φ = 0.17) fluorophore. While H_2_O_2_ was added into the **B2** system, the system emitted strong fluorescence at 500 nm.

The molar absorption coefficient and Stokes shift of **B2** and compound **1** had been studied in different solvents (Table S1). In organic solvents such as DCM, MeOH, MeCN, DMSO, DMF, and THF, **B2** and compound **1** showed a high molar absorption coefficient and large Stokes shift in MeCN; thus, MeCN was chosen as the mixture for further spectroscopic study. As shown in Figure 2b, the **B2** concentration was 5 µM, and the tested H_2_O_2_ concentrations ranged from 0 µM to 1000 µM. As the concentration of H_2_O_2_ increases, the fluorescence intensity at 500 nm gradually increases. Moreover, a linear relationship exists between the absorbance values at 500 nm (R^2^ = 0.9927). The LOD was calculated to be 49.74 nM, comparable to the reported detection methods for peroxynitrite (Table S3). These preliminary in vitro experiments indicated that probe **B2** acted as a good “turn-on” response sensor for determining H_2_O_2_.

### 2.3. AIE Property

**Figure 3:**
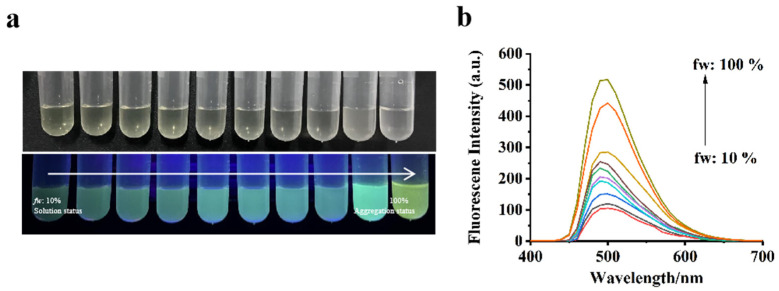
(**a**) Photographs of compound **1** in water/MeCN mixtures taken under natural light and UV illumination; (**b**) fluorescence spectra of compound **1** in the water/MeCN mixture. The experiments were repeated three times, and the data are shown as mean (±S.D.).

To further investigate the AIE property of compound **1**, the water/MeCN mixtures were used to estimate the influence of the water’s volume fraction (fw) on the fluorescence. The photo of compound **1** in different proportions of the water/MeCN solution under white light and ultraviolet light is shown in Figure 3a. When fw was increased, a distinct enhancement of fluorescence was observed, and the compound **1** solution emitted bright green fluorescence under a 365 nm UV lamp. The fluorescence intensity at 500 nm (λex = 325 nm) increased significantly with the increase in fw (Figure 3b). The change in fluorescent emission with the water fraction indicated that compound **1** has AIE characteristics.

### 2.4. Characteristics of H_2_O_2_ Sensing via Probe **B2**

**Figure 4:**
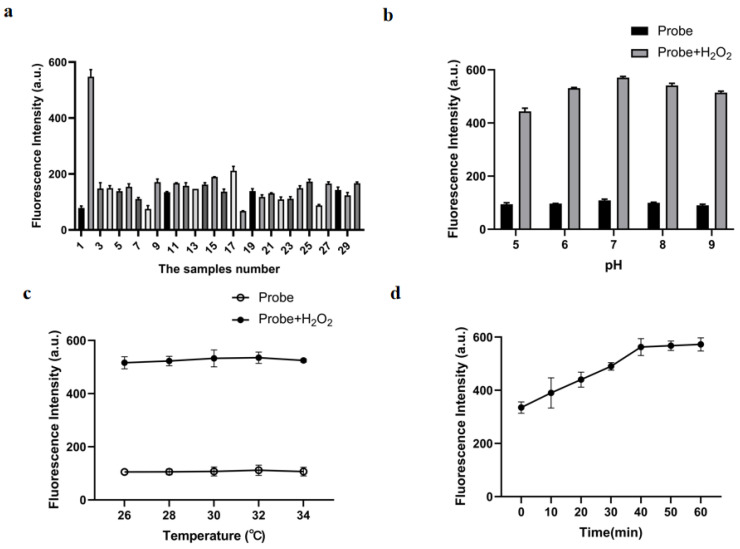
(**a**) Comparison of the fluorescence intensity of probe **B2** (5 μM) at 500 nm after incubation with (1) blank, (2) H_2_O_2_, (3) ONOO^−^, (4) O_2_^−^, (5) HOCl, (6) ^1^O_2_, (7) GSH, (8) TBHP, (9) Al^3+^, (10) Fe^3+^, (11) Fe^2+^, (12) Cu^2+^, (13) Ba^2+^, (14) Ca^2+^, (15) Mg^2+^, (16) K^+^, (17) Na^+^, (18) F^−^, (19) N_3_^−^, (20) HPO_4_^2−^, (21) HSO_4_^−^, (22) SO_4_^2−^, (23) HCO_3_^−^, (24) CO_3_^2−^, (25) CH_3_COO^−^, (26) OH^−^, (27) CN^−^, (28) NO_3_^−^, (29) NO_2_^−^, and (30) Cl^−^. Interfering substances were at a concentration of 500 μM, H_2_O_2_ (200 μM); (**b**) fluorescence spectral changes of **B2** at 500 nm before and after the addition of H_2_O_2_ at different pH; (**c**) **B2** fluorescence intensity with different temperatures; and (**d**) time-dependent curve of probe **B2** at 500 nm toward H_2_O_2_. The experiments were repeated three times, and the data are shown as mean (±S.D.).

In addition to H_2_O_2_, various active oxygen species and oxidants were present in organisms. To investigate the selectivity and anti-interference capability of probe **B2**, this study performed interference experiments on **B2** by introducing other interfering substances, including various ROS (H_2_O_2_, ONOO^−^, O_2_^−^, HOCl, ^1^O_2_, GSH, and TBHP), Al^3+^, Fe^3+^, Fe^2+^, Cu^2+^, Ba^2+^, Ca^2+^, Mg^2+^, K^+^, Na^+^, F^−^, N_3_^−^, HPO_4_^2−^, HSO_4_^−^, SO_4_^2−^, HCO_3_^−^, CO_3_^2−^, CH_3_COO^−^, OH^−^, CN^−^, NO_3_^−^, NO_2_^−^, Cl^−^, and a blank. Results are shown in Figure 4a. These similar interferences did not cause significant fluorescence changes in probe **B2** and did not affect the H_2_O_2_ recognition of probe **B2**. Only the incubation with H_2_O_2_ could induce the significant fluorescence enhancement of probe **B2**, while under the same conditions, no fluorescence increase could be detected for other potential interference.

The acid–base conditions may affect the stability and responsiveness of probe **B2** towards H_2_O_2_. Therefore, this experiment detected the fluorescence emission of probe **B2** before and after H_2_O_2_ addition at pH 5 to 9. As shown in Figure 4b, with the addition of H_2_O_2_, the probe **B2** fluorescence intensity was enhanced at pH 5 to 9, and the fluorescence intensity was maximal at pH 7. Given that the physiological pH value is about 7.4 and probe **B2** showed a favorable fluorescence response to H_2_O_2_ at pH 7, probe **B2** could be utilized for detecting H_2_O_2_ in living systems. Furthermore, Figure 4c illustrates the temperature-dependent fluorescence changes of the probe. The results showed that, with the addition of H_2_O_2_, the probe reached a plateau in the range of 23 to 36 °C. The fluorescent probe has good stability. Hence, the synthetic probe **B2**, developed in this experiment, holds promise for detecting H_2_O_2_ in the internal environment of living cells.

The time dependence of probe **B2** for detecting H_2_O_2_ was evaluated by measuring the fluorescence of **B2**. The time response of probe **B2** (5.0 μM) for H_2_O_2_ (200 μM) was performed (Figure 4d). After adding 200 μM of H_2_O_2_, the fluorescence intensity of probe **B2** increased with time until it reached a maximum at 40 min and remained stable. The experimental results showed that **B2** had the ability to detect H_2_O_2_ in real-time and could complete the detection within 40 min, while the fluorescence intensity almost no longer changed over time.

### 2.5. Cytotoxicity and Cell Imaging of **B2**

**Figure 5:**
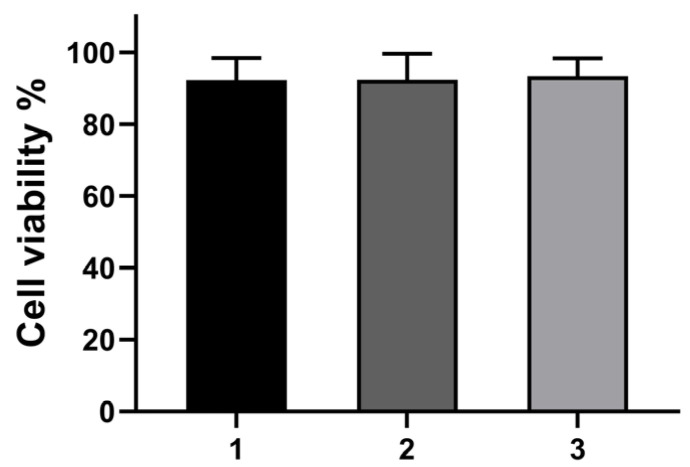
The cell survival rate of A549 cells. (**1**) After 5 min of irradiation with blue light; (**2**) after adding 30 µM of probe **B2**; and (**3**) after irradiation under blue light for 5 min and the addition of probe **B2**. The experiments were repeated three times, and the data are shown as mean (±S.D.).

The cytotoxicity of the probe was evaluated via the MTT assay using A549 cells. The test results are shown in Figure 5. At a probe **B2** concentration of 30 μM, the cell survival rate exceeded 90%. The IC_50_ values of the probe on A549 cells were all above 30 μM, indicating that they had no toxicity to normal cells at this concentration. Cells were seeded onto 96-well cell-culture plates and then irradiated for 5 min under the blue channel. The survival of cells was measured using a microplate reader, and the cell survival rate was 90%, 92%, and 98%, respectively. The cytotoxicity test confirmed the safety and reliability of the compounds obtained as chemical diagnostic drugs to a certain extent and established a foundation for subsequent experiments.

**Figure 6:**
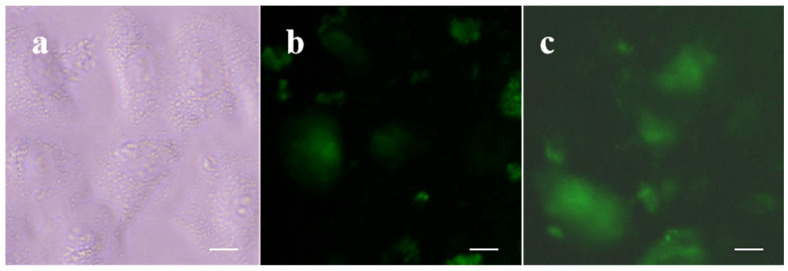
(**a**) Image of endogenous H_2_O_2_ in A549 cells untreated; (**b**) images of the probe and the PMA in A549 cells; and (**c**) images of probe **B2** and the exogenous H_2_O_2_ in A549 cells. Scale bar = 40 μm.

Investigating the physical properties of probe **B2**, further evaluations of its potential applications for localizing hydrogen peroxide in living cells have been conducted in the experiment below. Based on in vitro experiments and cytotoxicity assessments, the optimal conditions were a temperature of 37 °C and a probe concentration of 30 µM was established. This experiment employed probe **B2** to detect H_2_O_2_ under physiological conditions in live A549 cells. As shown in Figure 6a, A549 cells themselves had no fluorescence (blank group). As shown in Figure 6b, a conspicuous green fluorescence signal was observed upon treatment with PMA for 30 min and subsequent incubation with the fluorescent probe **B2** for 30 min. The above results showed that probe **B2** could detect endogenous H_2_O_2_. Figure 6c shows that these cells exhibited substantial fluorescence emission upon the addition of exogenous H_2_O_2_. Fluorescence imaging confirmed the ability of the probe **B2** fluorescence probe to detect both endogenous and exogenous H_2_O_2_.

## 3. Materials and Methods

### 3.1. Chemicals and Instruments

The ^1^H NMR and ^13^C NMR spectra were acquired at 25 °C in a nuclear magnetic resonance spectrophotometer (DRX—400, Bruker, Germany) at 400 MHz for ^1^H NMR in DMSO-*d*_6_ and 100 MHz for ^13^C NMR in DMSO-*d*_6_. Thin-layer chromatography (TLC) was performed on silica gel (GF254, Qingdao Haiyang Chemical Co., Ltd., Qingdao, China) with a visualization of the spots under UV light at 254 nm. Fluorescence spectra used a fluorescence spectrophotometer (F-2710, Hitachi, Hiroshima-shi, Japan). Absorption measurements were conducted using a UV–Vis spectrophotometer (UH5300, Hitachi, Japan) within a 190–1100 nm wavelength range.

### 3.2. Synthesis of the Fluorescence Probe **B2**

#### 3.2.1. Synthesis of (E)-2-(Imidazole[1,2-a] Pyridin-2-yl) Phenol (**1**)

NaHCO_3_ (126 mg, 1.5 mmol) was added to the ethanol solution (15 mL) of a solution of 2-hydroxyacetophenone (642.0 mg, 3.0 mmol) and 2-aminopyridine (282.2 mg, 3.0 mmol). The resulting mixture was refluxed for 2 h, and the reaction was monitored using thin-layer chromatography (TLC). After completion of the reaction, the formed precipitate was filtrated and washed with 15 mL of acetone. The mixture was extracted with dichloromethane (3 × 20 mL). The combined organic layer was dried over MgSO_4_, and dichloromethane was evaporated under reduced pressure to give compound **1** (458.6 mg, 72.8%). ^1^H NMR (400 MHz, DMSO-*d*_6_) δ 12.05 (s, 1H, OH), 8.62 (dd, 1H, *J* = 4.0 Hz, ArH), 8.50 (s, 1H, ArH), 7.89 (dd, 1H, *J* = 8.0 Hz, *J* = 4.0 Hz, ArH), 7.68 (d, 1H, *J* = 8.0 Hz, ArH), 7.35 (t, 1H, *J* = 8.0 Hz, ArH), 7.20 (1H, t, *J* = 8.0 Hz, ArH), 7.00 (t, 1H, *J* = 8.0 Hz, ArH), 6.93 (d, 1H, *J* = 8.0 Hz, ArH), and 6.89 (d, 1H, *J* = 8.0 Hz, ArH). ^13^C NMR (100 Hz, DMSO-d6) δ 156.6, 143.5, 143.4, 129.6, 127.3, 127.0, 126.2, 119.6, 116.4, 113.4, and 109.8. HRMS (ESI-TOF) C_13_H_10_N_2_O [M + H]^+^ 211.0827, found 211.0876.

#### 3.2.2. Synthesis of (E)-2-(2-((4-(4,4,5,5-tetramethyl-1,3,2-dioxaborolan-2-yl) Benzyl) oxy) Phenyl) Imidazo [1,2-a] Pyridine (**B2**)

K_2_CO_3_ (239.8 mg, 2.2 mmol) was added to the anhydrous acetonitrile (10 mL) solution of **1** (420 mg, 2.0 mmol) and 4-(Bromomethyl) benzene boronic acid pinacol ester (651.2 mg, 2.2 mmol). The mixture was heated to reflux for 6 h and then cooled to room temperature. A white solid was produced, filtered, and recrystallized with DMC and hexyl hydride to isolate the purified product **B2** (624.8 mg, 73.3%). ^1^H NMR (400 MHz, DMSO-*d*_6_) δ 8.49 (dd, 1H, *J* = 8.0 Hz, *J* = 1.6 Hz, ArH), 8.16 (s, 1H, ArH), 8.02 (d, 1H, *J* = 8.0 Hz, ArH), 7.88 (d, 2H, *J* = 8.0 Hz, ArH), 7.61 (d, 1H, *J* = 8.0 Hz, ArH), 7.53 (d, 2H, *J* = 8.0 Hz, ArH), 7.28–7.32 (m, 1H, ArH), 7.13–7.17 (m, 1H, ArH), 7.05 (d, 1H, *J* = 8.0 Hz ArH), 6.73 (t, 1H, *J* = 8.0 Hz, ArH), 5.25 (2H, s, ArH), and 1.39 (s, 12H, CH_3_). ^13^C NMR (100 Hz, DMSO-*d*_6_) δ 155.6, 144.0, 141.0, 140.6, 135.2, 128.9, 127.5, 127.2, 125.4, 122.9, 121.3, 116.8, 113.3, 113.0, 112.3, 83.9, 70.0, and 24.9. HRMS (ESI-TOF) C_26_H_27_BN_2_O_3_ [M + H]^+^ 426.3230, found 427.2193.

### 3.3. Spectroscopic Materials and Methods

The fluorescence intensity of fluorescent probe **B2** measurement experiments was conducted in a 10% MeCN solution (MeCN/H_2_O = 1:9, *v*/*v)*. The fluorescent emission spectra were recorded at 350 to 650 nm.

### 3.4. Measurement of the Detection Limit

The detection limit was obtained from the fluorescence titration curve and was calculated according to the following equations: Detection limit = 3σ/k. Here, σ represents the deviation of the blank, and k is the slope of the linear regression equation.

### 3.5. Cytotoxic Assay

Dulbecco’s modified Eagle’s medium (DMEM) was used to culture A549 cells at 37 °C. The MTT assay was used to measure the cytotoxicity of probe **B2** towards A549 cells. About 5 × 10^4^ cells/well were seeded onto 96-well cell-culture plates for 24 h. Then, different concentrations of probes were added to the wells. After another 12 h, the MTT (100 µL) was added to each well and incubated at 37 °C under 5% CO_2_ for 4 h. The MTT solution was removed, purple precipitates (formazan) observed in plates were further dissolved in DMSO (100 µL), and a microplate reader was used to measure the absorbance for each well.

### 3.6. Cell Image Experiments

The A549 cells were cultured in a DMEM culture medium supplemented with 1% (vol/vol) penicillin and 10% (vol/vol) fetal bovine serum at 37 °C in a 5% CO_2_ atmosphere for 24 h. Before the experiment, the cell culture medium was poured off, washed three times with PBS, and changed into a new medium. For the imaging of cellular endogenous H_2_O_2_, A549 cells were incubated with 100 µM H_2_O_2_ for 30 min in the control group. In the experimental group, one group of A549 cells was treated with the probe alone for 30 min. The ROS levels in A549 cells were upregulated using the activator PMA (phorbol-12-myristate-13-acetate, 1 μM) for 30 min. In the other group, A549 cells were pretreated with PMA for 30 min and then incubated with the probe for an additional 30 min. To detect exogenous H_2_O_2_, cells were first incubated with H_2_O_2_ for 30 min, and then A549 cells were treated with a probe **B2** for 30 min. Finally, 1 mL of paraformaldehyde was added to each group of cells and fixed for 5 min. After three washes with PBS, 1 mL DAPI dye (1 µg/mL) was added to each well, nuclear staining for 1–5 min, and finally washed three times with PBS. Finally, a fluorescence inverted microscope was applied to fluorescence imaging.

## 4. Conclusions

In summary, the 2-(2-((4-(4,4,5,5-tetramethyl-1,3,2-dioxaborolan-2-yl) benzyl) oxy) phenyl) imidazo[1,2-a] pyridine (**B2**) had been designed and synthesized successfully for the first time to detect H_2_O_2_ in living cells. It can react with H_2_O_2_ selectively, thus producing a green fluorescence signal at 500 nm. The probe **B2** demonstrates high selectivity, sensitivity, and anti-interference ability. Importantly, the synthesis process of **B2** was simple, and the yield was high. This study has successfully employed probe **B2** to visualize H_2_O_2_ in A549 cells, allowing clear and visual monitoring of both exogenous and endogenous changes in H_2_O_2_ levels. It is expected that **B2** will become a promising tool to detect H_2_O_2_ and exhibit important potential applications in the diagnosis of H_2_O_2_-related diseases.

## Data Availability

Data are contained within the article.

## Supplementary Materials

The following supporting information can be downloaded at: https://www.mdpi.com/article/10.3390/molecules29040882/s1, Figures S1–S6: ^1^H NMR, ^13^C NMR and MS of all products; Figure S7: UV–Vis response of **B2** towards H_2_O_2_; Table S1: The photoluminescence properties of **B2** and compound **1**; Table S2: The microscope set-up (for the imaging experiments); Table S3: Comparing the limit of detection with other reported methods for H_2_O_2_ detection. CheckCIF report of compound **1**.
