# Supplementary material for: Design, Synthesis of Hydrogen Peroxide Response AIE Fluorescence Probes Based on Imidazo [1,2-a] Pyridine
Source: Molecules. 2024 Feb 16;29(4):882. doi: 10.3390/molecules29040882 (PMC10891862; doi:10.3390/molecules29040882)
Supplement: Supplementary file 1 [file molecules-29-00882-s001.zip › Supporting Information.pdf]

Supporting information

Design, synthesis of hydrogen peroxide response AIE fluorescence probes based on imidazo [1,2-a] pyridine

Luan Tong [1], Yulong Yang [2], Likang Zhang [1], Jiali Tao [2],Bin Sun [2], Cairong Song [1],Mengchen Qi [1], Fengqing Yang [2], Mingxia Zhao [2,3,*], and Junbin Jiang [1, 2,*]

1 Department of Veterinary Medicine, Shanxi Agricultural University, Jinzhong 030801, China
2 Department of Mining Engineering, Shanxi Institute of Technology, Yangquan 045000, China
3 Yangquan technology innovation center of Carbon dioxide capture, utilization and storage, Shanxi Institute of Technology, Yangquan 045000, China
* Correspondence: jiangjunbing@sxit.edu.cn (J.B. Jiang), zhmx@sxit.edu.cn (M.X. Zhao).

contents:

1. ^{1}H-NMR of target compounds

2. ^{13}C-NMR of target compounds

3. Mass Spectrum (MS) of target compounds

4. Fluorescence emission spectra of compounds

5. The photophysical properties of B2 and compound 1

6. The microscope set-up (for the imaging experiments)

7. Comparison of fluorescent probes for hydrogen peroxide

1.^{1}H-NMR of target compound **1**

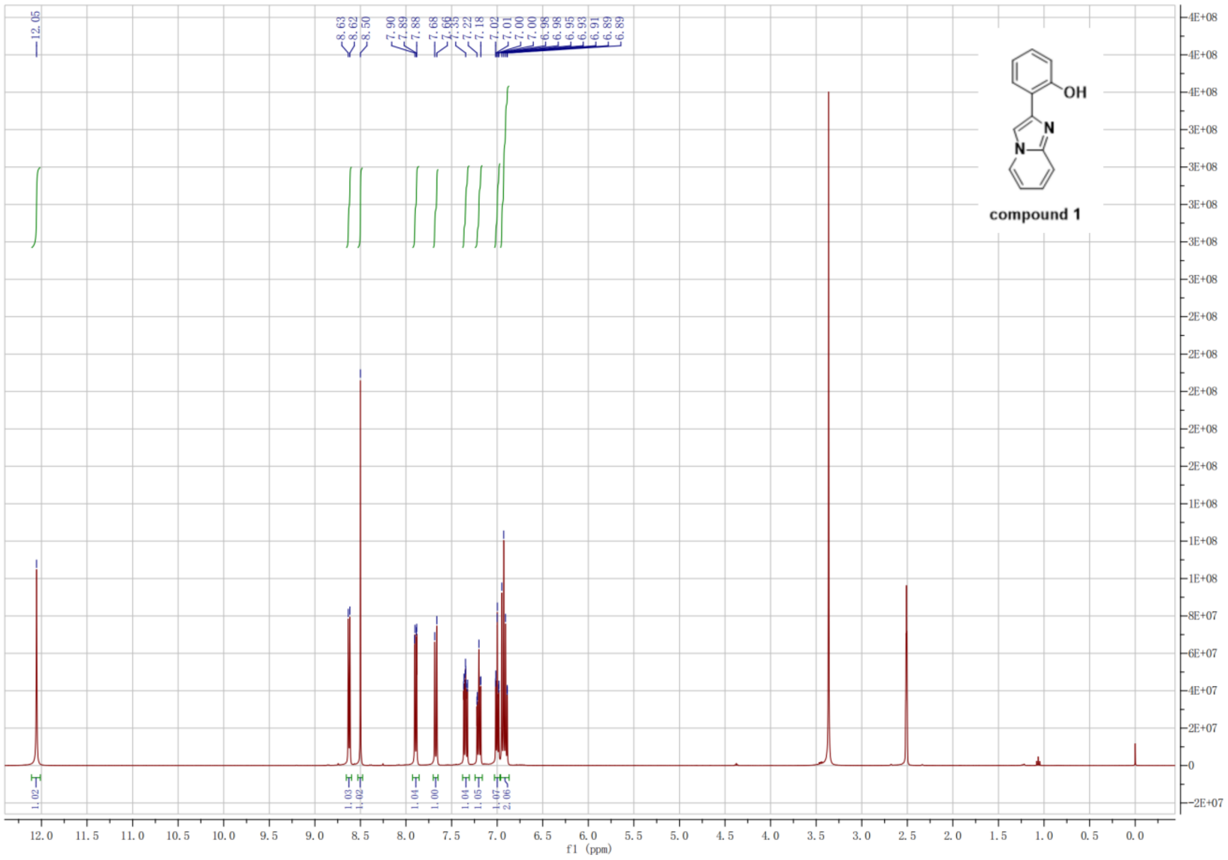

Fig. S1: ^{1}H NMR for compound **1**

1.^{1}H-NMR of target compound **B2**

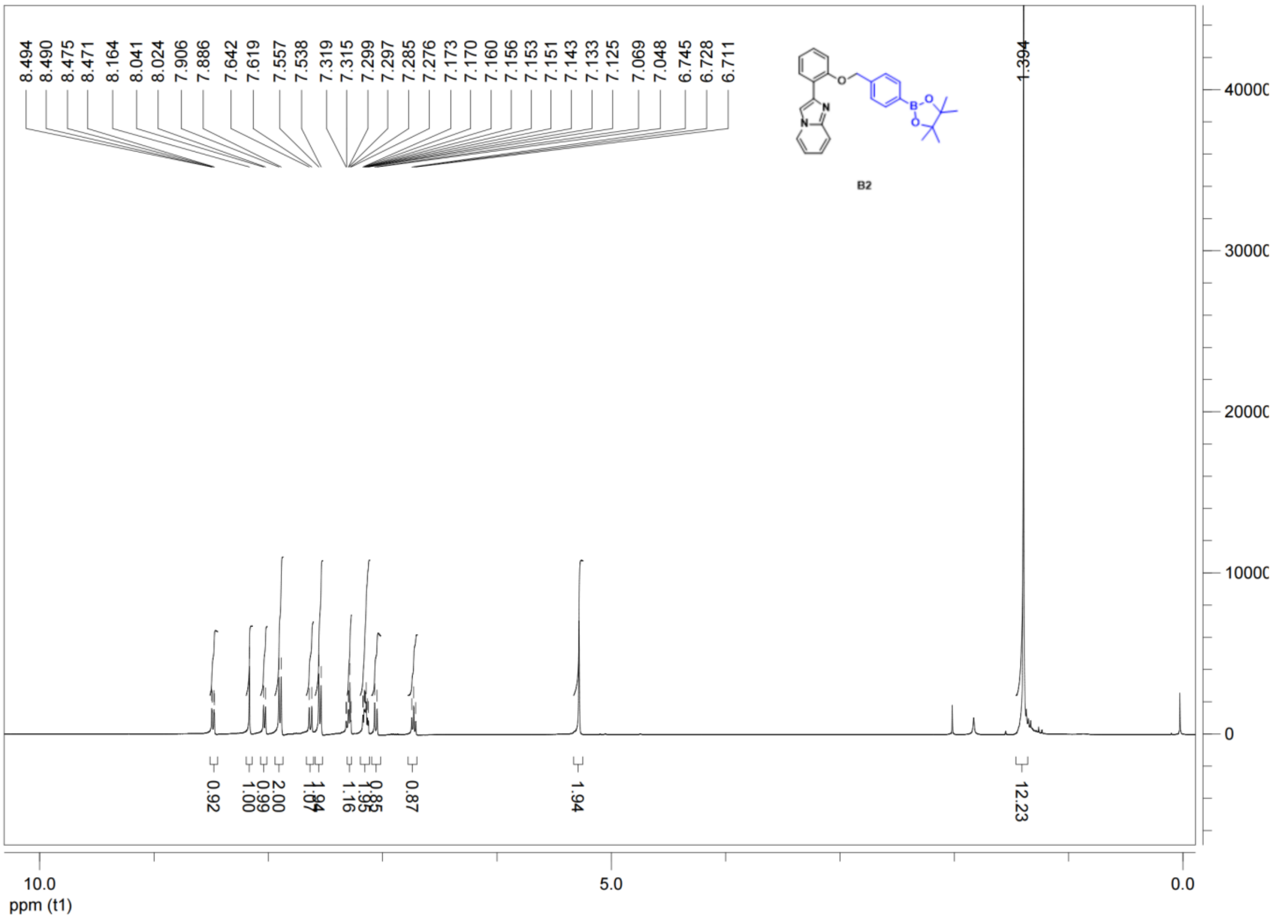

Fig. S2: ^{1}H NMR for compound **B2**

2. ^{13}C-NMR of target compound **1**

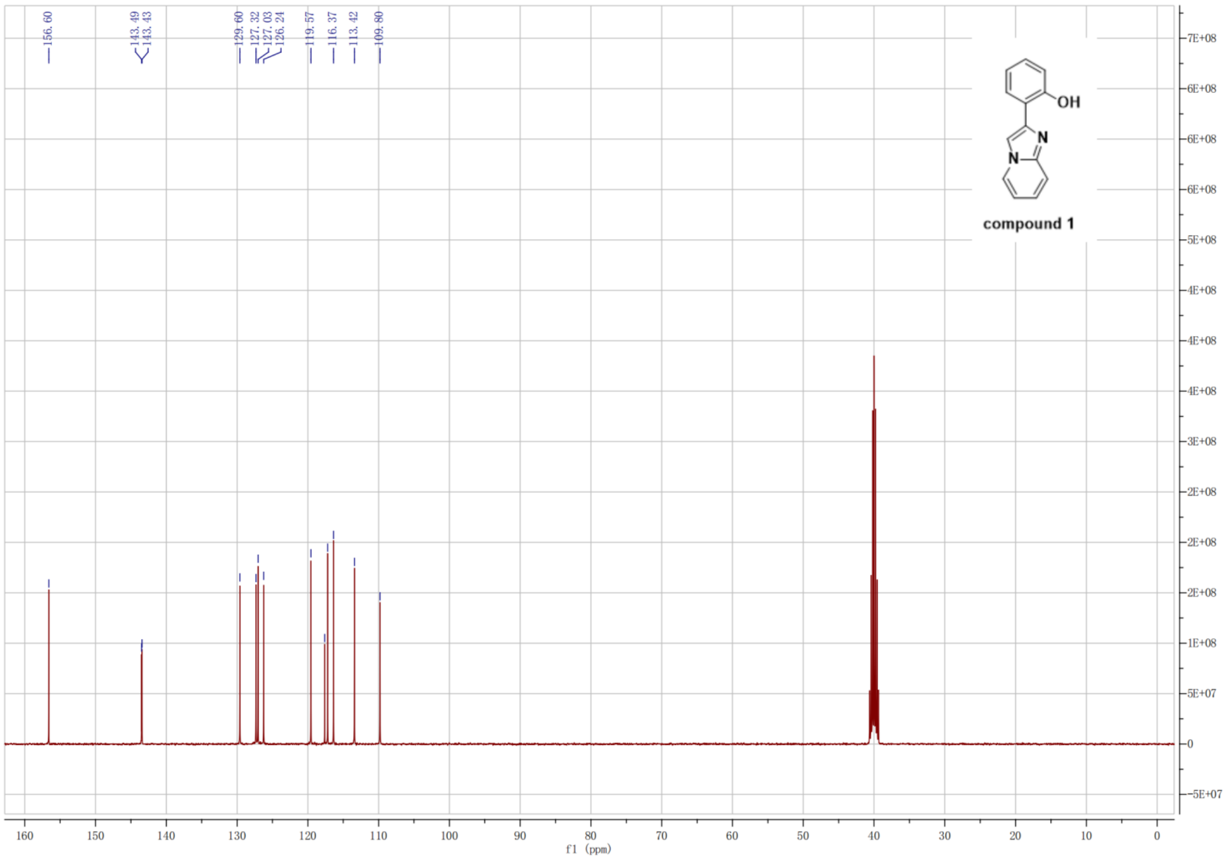

Fig. S3: ^{13}C NMR for compound **1**

2. ^{13}C-NMR of target compound **B2**

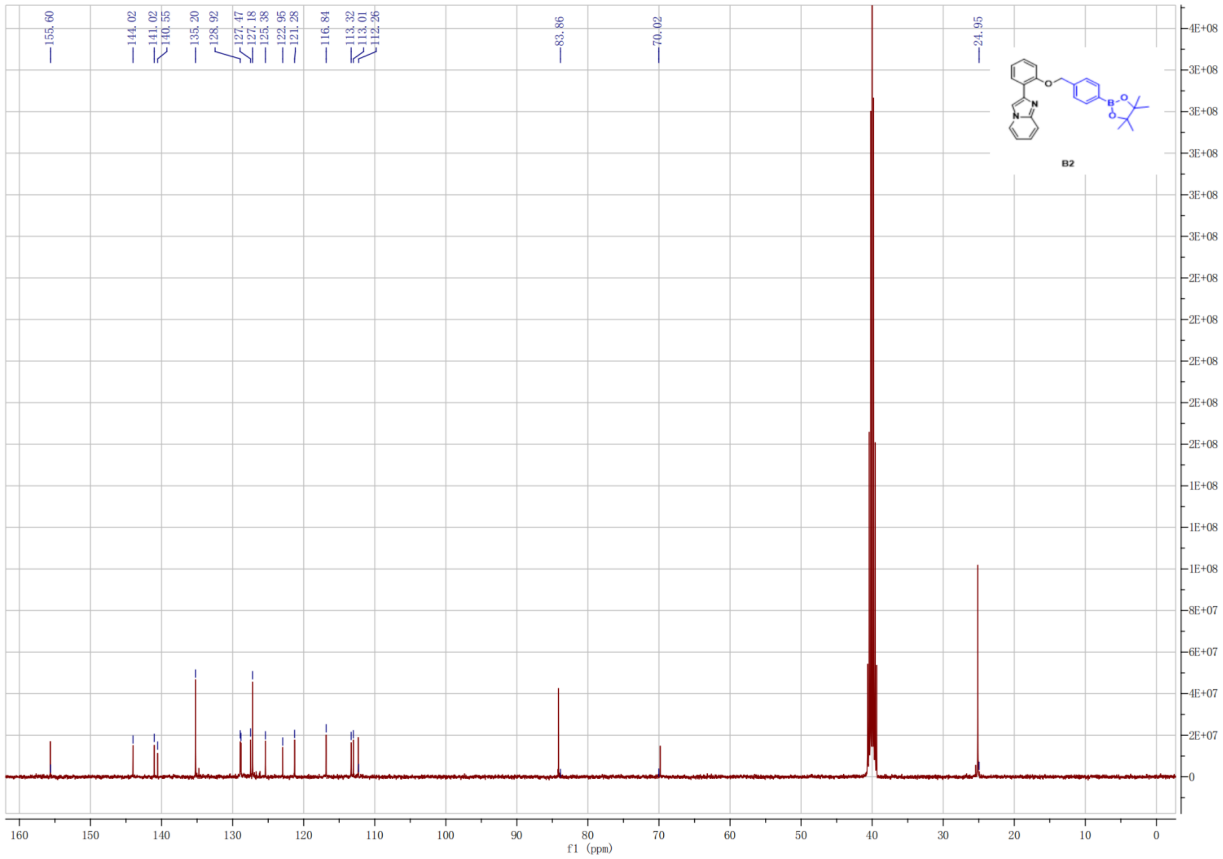

Fig. S4: ^{13}C NMR for compound **B2**

3. MS of target compound **1**

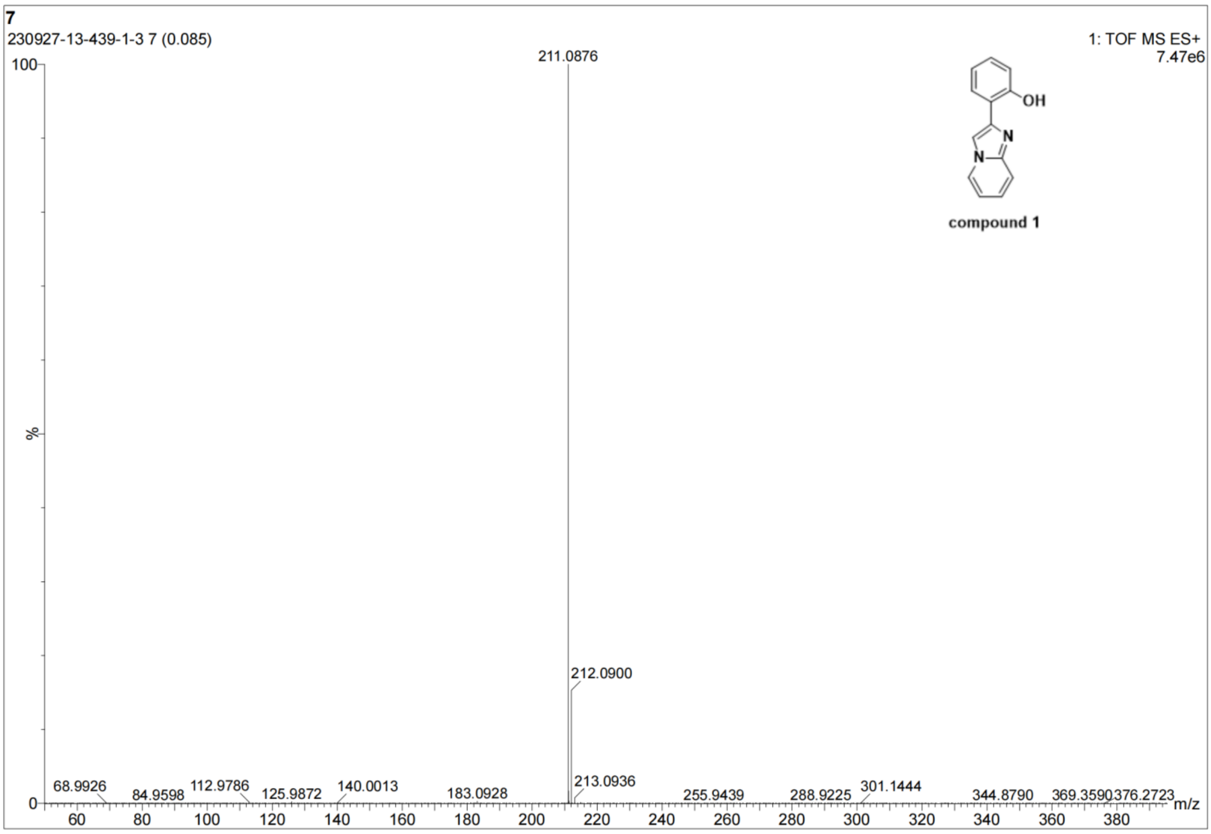

Fig. S5: MS for compound **1**

3. MS of target compound **B2**

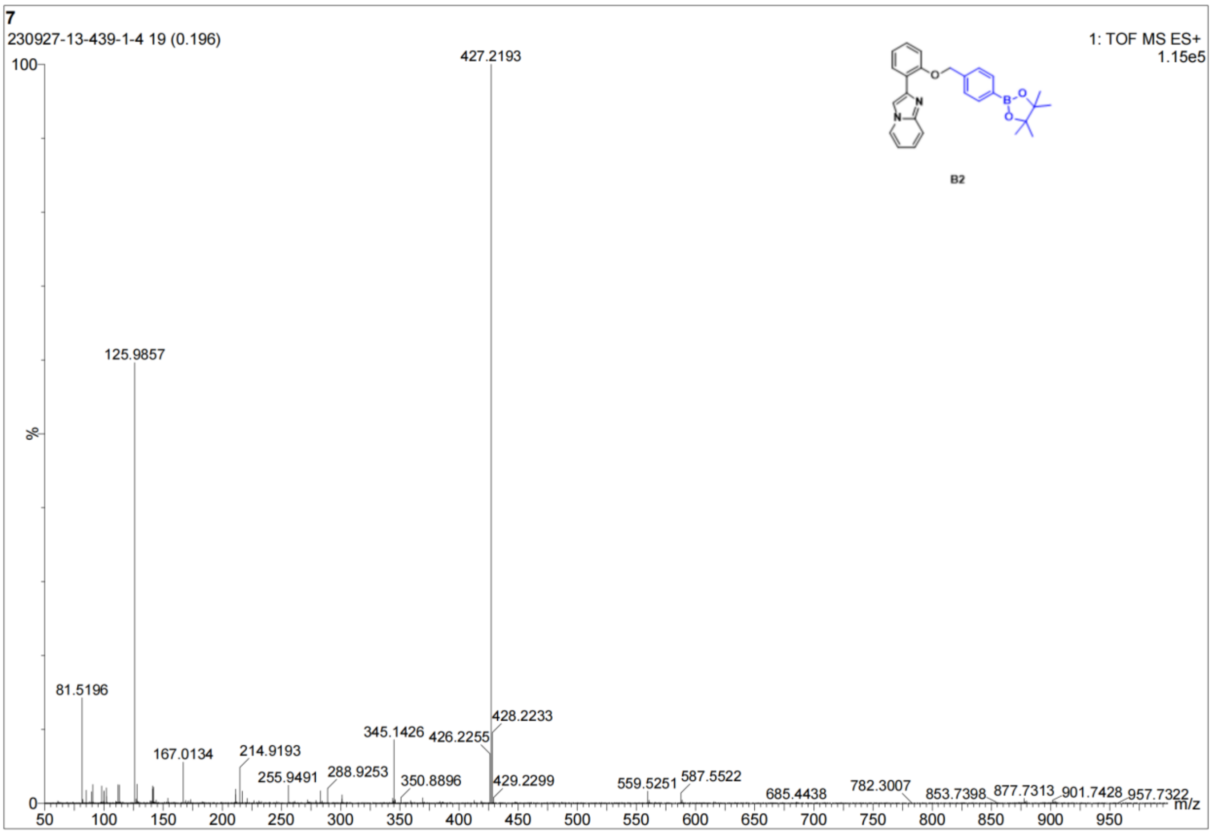

Fig. S6: MS for compound **B2**

4.Fluorescence emission spectra of compounds

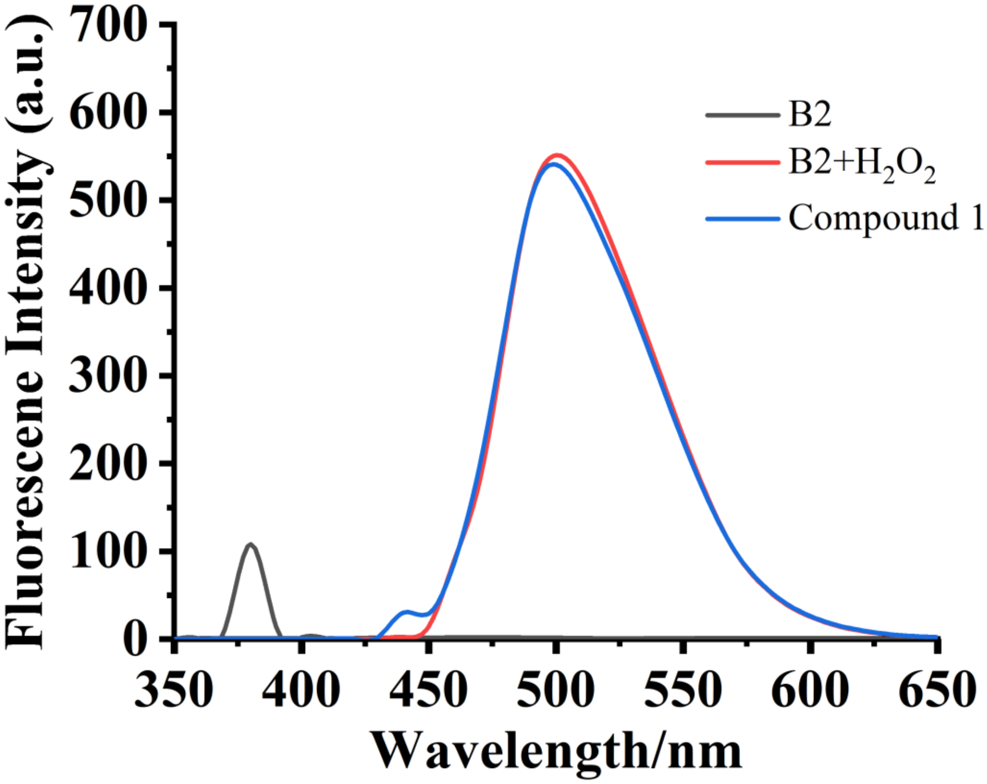

Fig. S7: Fluorescence emission spectra of compound **1**, probe **B2** before and after addition of H_2O_2.

5.The photoluminescence properties of **B2** and compound **1**

Table. S1: The photophysical properties of **B2** and compound **1**

**Solvent**	**Solvent**	**λ_{Abs} [a] (nm)**	**λ_{em} [b] (nm)**	**Stokes shift (nm)**	**Ɛ(×10^4) (L·mol^{-1}·cm^{-1})**
**B2**	DCM	325	385	60	2.558
**B2**	MeOH	320	375	55	2.846
**B2**	MeCN	314	395	80	3.164
**B2**	DMSO	329	391	62	3.132
**B2**	DMF	316	384	68	2.938
**B2**	THF	326	385	59	3.164
**Compoung 1**	DCM	325	505	180	8.530
**Compoung 1**	MeOH	330	495	165	8.336
**Compoung 1**	MeCN	318	506	187	9.116
**Compoung 1**	DMSO	310	490	180	7.800
**Compoung 1**	DMF	326	505	179	8.440
**Compoung 1**	THF	315	492	177	8.001

[a]Maximum absorption wavelength (nm). [b]Maximum emission wavelength (nm).

6.The microscope set-up (for the imaging experiments)

Table S2: The microscope set-up (for the imaging experiments)

**Model**	Nikon Ts2-FL
**Light filter used**	Blue bandpass filters
**Excitation wavelength**	470 nm
**Power**	30 W
**Time frame**	10 ms - 2 s

7.Comparison of fluorescent probes for hydrogen peroxide

Table S3: Comparing the limit of detection with other reported methods for H_2O_2 detection.

Probes	Stokes shift	Limit of detection	Response time	Application	synthesis steps	Reference
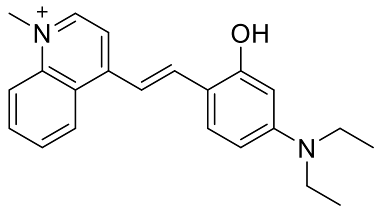	105nm	1.2μM.	30 min	HepG2	2steps	Talanta, 217, (*2020*) 121000.
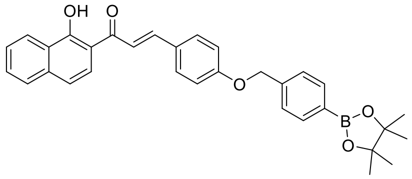	189nm	78.89nM	3.4min	U87	3steps	Sensors and Actuators B-chemical. 248 (2017) 257-264.
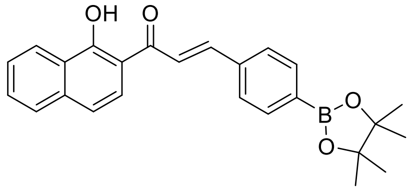	145nm	58.13nM	10min	U87	1steps	Sensors and Actuators B-chemical. 248 (2017) 257-264.
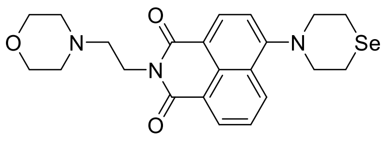	135nm	61 mM	60min	MCF-7	2steps	Spectrochimica Acta Part A: Molecular and Biomolecular Spectroscopy. 276

						(2022) 121218-121218.
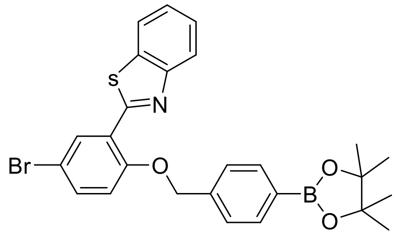	128nm	132 nM	40min	MCF-7	2steps	Bioorganic Chemistry. 123 (2022) 105798-105798.
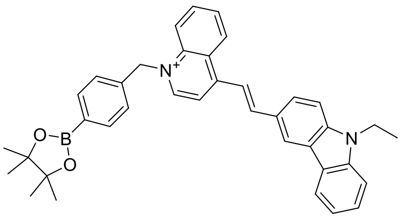	151nm	0.04 μM	4min	Hela	4steps	Analytical Chemistry. 88 (2015) 1455-1461.
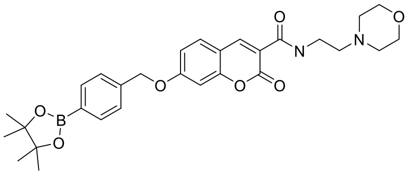	100nm	0.13 μM	20.528 min	MCF-7	3steps	Molecules. 26 (2021) 3352-3352.
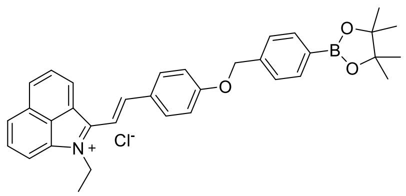	133nm	67 nM	30min	Hela	2steps	Chinese Journal of Organic Chemistry. 40 (2020) 2888-2888.
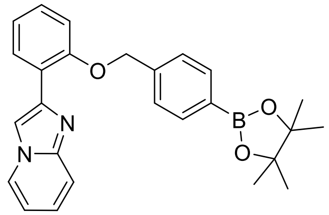	187nm	49.74 nM	45min	A549	2steps	This work
